# Promoter hypomethylation associated with elevated *TRIM33* expression in triple-negative breast cancer patients

**DOI:** 10.3389/fonc.2026.1973520

**Published:** 2026-10-30

**Authors:** Archana Chaudhary, Manish Kumar, Md. Jawed Akhtar, Sheersh Massey, Lalit Mohan, Md. Margoob Ahmad

**Affiliations:** 1 Department of Pharmacology, Indira Gandhi Institute of Medical Sciences, Patna, Bihar, India; 2 Department of Surgical Oncology, Indira Gandhi Institute of Medical Sciences, Patna, Bihar, India; 3 Department of Anatomy, Indira Gandhi Institute of Medical Sciences, Patna, Bihar, India; 4 Dr. RP Centre for Ophthalmic Sciences, All India Institute of Medical Sciences, New Delhi, India

**Keywords:** BC (breast cancer), cancer biomarker, TGF-β, TNBC (triple negative breast cancer), *TRIM33*

## Abstract

**Introduction:**

Triple-negative breast cancer (TNBC) is a highly aggressive and diverse subtype of breast cancer with few therapeutic choices and poor prognosis. This is mostly because of recurrence, metastasis, and therapeutic resistance. Members of the tripartite motif-containing protein family (TRIM) have emerged as significant regulators of tumor growth due to their functions in cell proliferation, invasion, migration, and metastasis. *TRIM33* has been shown to operate context-dependently as either a tumor suppressor or a tumor promoter in various cancers, partly because of its participation in TGF-β-related signaling pathways. However, little is known about the expression pattern and epigenetic regulation of *TRIM33* in TNBC, especially in Indian patients.

**Methods:**

Twenty-eight patients with histologically confirmed TNBC were included in the study. Tumor tissue and paired adjacent non-tumor breast tissue were collected at the time of surgery. *TRIM33* mRNA and protein expression were evaluated using quantitative real-time PCR (qRT-PCR), western blotting, and immunohistochemistry. *TRIM33* promoter methylation status was assessed using methylation-specific PCR (MS-PCR).

**Results:**

*TRIM33* expression was increased in TNBC tissue compared with paired adjacent non-tumor tissue. Increased *TRIM33* mRNA expression was observed in 19 of 28 cases (67.9%), while elevated TRIM33 protein expression was detected in 16 of 28 cases (57.1%). An unmethylated/hypomethylated *TRIM33* promoter pattern was observed in 22 of 28 cases (78.6%).TRIM33 protein expression showed significant associations with selected clinicopathological characteristics, including clinical stage. Promoter methylation status was also associated with selected clinicopathological parameters.

**Conclusion:**

Promoter hypomethylation of *TRIM33* was frequently observed in TNBC and was associated with increased *TRIM33* expression and selected clinicopathological characteristics. These findings suggest a potential role for epigenetic regulation of *TRIM33* in TNBC and warrant validation in larger patient cohorts.

## Introduction

Breast cancer is one of the most prevalent cancers that affect women worldwide. Human epidermal growth factor receptor 2 (HER2), progesterone receptor (PR), and estrogen receptor (ER) negativity are characteristics of TNBC; these receptors are frequently found in other breast cancer subtypes (1). TNBC is an aggressive subtype of breast cancer associated with high molecular heterogeneity, increased metastatic potential, and limited targeted treatment options, contributing to its unfavorable clinical outcomes. The primary treatment available for TNBC is chemotherapy; however, the overall survival probability of TNBC patients is greatly reduced due to drug resistance and the lack of universal and well-defined molecular targets (2). The molecular heterogeneity of TNBC contributes to considerable intertumoral and intratumoral differences in tumor progression, metastatic potential, prognosis, and therapeutic response (3). Consequently, identifying epigenetically regulated molecular alterations, including aberrant promoter methylation associated with altered gene expression, may help define biologically distinct subsets of TNBC and contribute to molecular stratification (3). Characterizing these epigenetic alterations may further enhance our understanding of TNBC biology and provide a basis for the future development and validation of candidate biomarkers and potential therapeutic targets.

The development of malignant tumors has been linked to aberrant Tripartite motif (TRIM) family protein expression, according to several studies (4). Tripartite-motif protein (TRIM) family proteins and ubiquitin E3 ligase activity are crucial for several cellular functions, such as autophagy, innate immunity, apoptosis, and cell growth (5). The protein-coding gene *TRIM33* (tripartite motif-containing 33), also known as transcriptional intermediary factor 1 gamma (*TIF1γ*), encodes a multifunctional protein involved in TGF-β/Smad signaling, transcriptional regulation, and cellular differentiation. In the present study, we performed CpG-island prediction within the 600-bp *TRIM33* promoter sequence analyzed for methylation-specific PCR. Using criteria of an island size >100 bp, GC content >50%, and an observed/expected CpG ratio >0.60, one CpG island was identified within the analyzed promoter region. The role of *TRIM33* in breast cancer biology has also been supported by recent evidence identifying *TRIM33* as an estrogen-dependent interactor and regulator of estrogen receptor-α (ERα) activity in ER-positive breast cancer (6). Importantly, previous studies have implicated *TRIM33* dysregulation and altered promoter methylation in cancer, providing a rationale for investigating epigenetic regulation of *TRIM33* in breast cancer. Therefore, deregulated activity or impaired gene expression of these factors is usually the etiology of cancer (7). Studies have demonstrated that certain TRIM protein members are linked to different types of cancer (8). According to reports, downregulated *TRIM33* enhances the ability of gastric cancer cells BGC-823 and SGC-7901 to multiply, form colonies, and migrate (9). Studies show that *TRIM33* activates transforming growth factor beta (TGF-β) signaling, which silences downstream genes and causes the *TRIM33-Smad2/3* complex to develop (10). However, the role of *TRIM33* in tumorigenesis remains controversial and appears to be highly context-dependent, with both tumor-promoting and tumor-suppressive functions reported across different malignancies (11). For instance, *TRIM33* is highly expressed in colorectal cancer, and its expression has been associated with tumor stage (12).Moreover, increased *TRIM33* expression in colorectal cancer has been linked to loss of *SMAD4* and is associated with poor clinical outcomes, suggesting that *TRIM33* may contribute to tumor progression in this specific molecular context (13).

Numerous complex genetic, epigenetic, and environmental factors affect the development and progression of TNBC (14). Many patients experience recurrence and metastasis after surgery, which is one of the primary causes of poor survival in TNBC. Therefore, it is essential to identify new molecular markers that capture the complex heterogeneity and aggressive nature of TNBC. However, the biological role of *TRIM33* and its expression pattern in human TNBC have not yet been fully understood. Epigenetic mechanisms, including DNA methylation and histone modifications, can alter the expression of tumor suppressor genes and oncogenes, thereby contributing to carcinogenesis. Therefore, we can gain a better understanding of the causes and mechanisms of cancer cell heterogeneity in TNBC by examining *TRIM33* epigenetic components. However, the expression pattern and epigenetic regulation of *TRIM33* in TNBC remain poorly understood. Therefore, the present study aimed to evaluate *TRIM33* expression at the mRNA and protein levels in TNBC and to examine its association with clinicopathological characteristics. We further investigated the methylation status of the *TRIM33* promoter to explore its possible relationship with altered *TRIM33* expression in TNBC.

## Materials and methods

### Sample collection

Tumor tissue and paired adjacent non-tumor breast tissue were collected from 28 female patients with histologically confirmed TNBC who underwent surgery at the State Cancer Institute, Indira Gandhi Institute of Medical Sciences, Patna, Bihar, India. The adjacent tissue was confirmed to be free of tumor involvement. Immediately after collection, the tissue samples were appropriately preserved for subsequent molecular analyses. Clinicopathological characteristics, including age, menopausal status, lymph-node status, tumor size, clinical stage, and tumor grade, were recorded for each patient. Only patients who were negative for ER and PR and did not meet the criteria for HER2 positivity were included in the present study. Patients with other breast cancer subtypes were excluded.

The study was approved by the Institutional Ethics Committee of the Indira Gandhi Institute of Medical Sciences (IGIMS), Patna, Bihar (Ref. No. 1268/IEC/IGIMS/2023). Written informed consent was obtained from all participants before enrollment in the study. The clinical, pathological, and demographic characteristics of the study participants are presented in **Table 1**.

**Table 1:** Clinicopathological characteristics of patients with triple-negative breast cancer (n = 28).

S. no	Patient group	Age (yr)	Stage	LN status	Tumor grading	Menopausal status
1.	P-1	35	III B	N1	2	Premenopausal
2.	P-2	49	III B	N2	2	Postmenopausal
3.	P-3	54	I A	N0	1	Postmenopausal
4.	P-4	41	III A	N1	2	Premenopausal
5.	P-5	38	III B	N2	2	Premenopausal
6.	P-6	50	III B	N1	2	Postmenopausal
7.	P-7	42	III A	N1	2	Premenopausal
8.	P-8	27	III A	N2	2	Premenopausal
9.	P-9	34	III B	N1	2	Premenopausal
10.	P-10	40	III B	N1	2	Premenopausal
11.	P-11	42	II A	N0	1	Premenopausal
12.	P-12	36	III B	N1	2	Postmenopausal
13.	P-13	42	III A	N2	1	Premenopausal
14.	P-14	67	III B	N2	2	Postmenopausal
15.	P-15	52	III B	N2	2	Postmenopausal
16.	P-16	34	II A	N1	2	Premenopausal
17.	P-17	55	III B	N2	2	Postmenopausal
18.	P-18	50	II A	N0	1	Postmenopausal
19.	P-19	56	III B	N1	2	Postmenopausal
20.	P-20	67	III B	N1	1	Postmenopausal
21.	P-21	56	III B	N1	2	Postmenopausal
22.	P-22	67	II A	N0	1	Postmenopausal
23.	P-23	48	III B	N2	2	Premenopausal
24.	P-24	52	III B	N2	2	Postmenopausal
25.	P-25	48	III B	N1	2	Premenopausal
26.	P-26	67	II A	N0	1	Postmenopausal
27.	P-27	62	III B	N1	2	Postmenopausal
28.	P-28	53	III B	N1	2	Postmenopausal

The table summarizes the demographic and clinicopathological characteristics of the enrolled patients, including age, menopausal status, tumor size, histological grade, lymph-node status, and clinical stage. Data are presented as number of patients (*n*) and percentage (%) unless otherwise indicated.

### Isolation of RNA and DNA

Total RNA and DNA were extracted from frozen tissue samples of TNBC patients using the Genomic DNA isolation kit (Qiagen, catalog no. 56304)and the total RNA Mini kit column technique (Qiagen, catalog ID 591105). The quality of the extracted nucleic acids was assessed using spectrophotometry (NanoDrop WPA, Biowave II, Germany). The integrity of the RNA and DNA was confirmed using agarose gel electrophoresis. To eliminate DNA contamination, total RNA was processed with DNase I using a Thermo Fisher Scientific kit (catalog no. 18068015). Using 1 μg of total RNA in 20 μL of total volume, cDNA synthesis was performed in accordance with the instructions in the kit (Thermo Fisher Scientific catalog No. 18090050).

### Quantitative real-time PCR analysis

Quantitative reverse transcription RT-qPCR analysis was conducted to evaluate the expression changes in *TRIM33* using a SYBR Green-based kit (ELK, Biotechnology, Catalog No-EQ001, Wuhan, China), according to the manufacturer’s instructions. *GAPDH* was used as the endogenous reference gene for normalization, and the corresponding matched adjacent non-tumor breast tissue was used as the calibrator. For each sample, ΔCt ​was calculated as ^Ct^(*TRIM33*) − ^Ct^(*GAPDH*) and ΔΔCt was calculated by subtracting the ΔCt ​of the matched adjacent non-tumor tissue from that of the corresponding tumor tissue. Relative *TRIM33* expression was calculated using the 2^−ΔΔCt^ method. The primer set used is detailed in **Supplementary File S1**. Each sample was analyzed in technical duplicate, and the mean Ct value of the two measurements was used for subsequent analysis. PCR was run in 40 cycles with denaturation at 94 °C for 45 seconds, annealing at 55 °C for 60 seconds, and extension at 72 °C for 60 seconds.

### Western blot

To extract total protein, the tissue samples were homogenized in RIPA (cell lysis buffer) (P0013B, Beyotime), and the concentration was determined using the BCA assay (Thermo Fisher Scientific Inc., USA, catalog-A65453).Thirty micrograms of total protein from each sample were separated by 10% SDS-PAGE and transferred onto PVDF membranes(IPFL00010, Millipore Sigma) and incubated overnight at 4 °C. Primary antibodies against *TRIM33* (1:1000, catalog no. D7U4F, Cell Signaling Technology, USA) and *GAPDH* (1:1000, catalog No.2118, Cell Signaling Technology, USA) were added to the blot after the membranes were blocked using skim milk. Additionally, at room temperature, secondary antibody incubation was conducted using the HRP-conjugated goat anti-rabbit IgG antibody (1:10,000,Thermo Fisher Scientific Inc., USA, Catalogue # A-11008) for 2 hours. ECL (cat: 170–5060) (Bio-Rad) was used for protein detection, and ImageJ was used for densitometry analysis. At least three separate experiments were conducted for each western blot assay.

### Immunohistochemistry

Immunohistochemistry was performed to evaluate TRIM33 protein expression using a *TRIM33*-specific antibody (#D7U4F, Cell Signaling Technology, USA). Formalin-fixed, paraffin-embedded breast tissue sections were mounted on poly L-lysine-coated slides, deparaffinized with xylene, and rehydrated with graded concentrations of ethanol. Endogenous peroxidase activity was blocked with 0.3% hydrogen peroxide, followed by antigen retrieval using citrate buffer. The sections were incubated overnight at 4 °C with the primary antibody at a dilution of 1:200, followed by incubation with the secondary antibody for 1 h at 37 °C. Antibody binding was visualized using 3,3′-diaminobenzidine (DAB) as a chromogenic substrate, and the sections were counterstained with hematoxylin. Normal breast tissue was used as a positive control, whereas sections processed without the primary antibody served as negative controls. Immunostaining was evaluated by an experienced histopathologist using a light microscope at ×40 and ×100 magnifications. Based on the percentage of positively stained tumor cells, *TRIM33* expression was categorized as follows: no expression (0%), mild expression (1–10%), moderate expression (>10–50%), and high expression (>50%).

### Methylation analysis by MS-PCR

Genomic DNA was bisulphite -converted using the EZ DNA Methylation-Lightning Kit (Zymo Research, Cat. No. D5030), according to the manufacturer’s instructions. The resulting bisulphite converted DNA was amplified by PCR using methylation-specific (M) and unmethylation-specific (U) primers targeting the *TRIM33* promoter. MS-PCR primers were custom-designed to specifically amplify the methylated and unmethylated sequences within this CpG-rich promoter region following bisulphite conversion. The primer sequences, amplicon sizes, genomic location of the targeted promoter region, and the number of CpG sites interrogated by the primer sets are provided in **Supplementary File S1**. The methylation-specific primers target the same promoter region and discriminate between methylated and unmethylated DNA based on bisulphite-conversion-dependent sequence differences. For the unmethylated and methylated positive controls, commercially available fully unmethylated and fully methylated human genomic DNA (Zymo Research, Orange, CA, USA) were used. PCR was performed in a total volume of 25 µL containing 1 U of Hot Start Taq DNA Polymerase (Qiagen; Cat. No. 203203), 0.5 µM each of the forward and reverse oligonucleotide primers, 1.5 mM MgCl_2_, 200 µM of each deoxynucleotide triphosphate (dNTP), 100 ng of bisulphite-converted DNA, and PCR buffer. The conditions for the PCR reaction were as follows:

10 minutes of initial denaturation at 95 °C, 35 cycles of denaturation at 95 °C for 45 seconds, annealing at 57.9 °C for 30 seconds, extension at 72 °C for 45 seconds, and a final extension at 72 °C for 7 minutes. The PCR products were separated by electrophoresis on a 2% agarose gel and visualized using a UV transilluminator. All experiments were repeated as an internal quality control, and the results showed no signs of distortion. The expected amplification products were 170 bp for the methylation-specific primer set and 171 bp for the unmethylation-specific primer set.

### Statistical analysis

Statistical analyses were performed using GraphPad Prism (version 5.0; GraphPad Software, San Diego, CA, USA). Continuous variables are presented as mean ± standard deviation (SD), unless otherwise specified. Comparisons of *TRIM33* expression between paired tumor and adjacent non-tumor tissues were performed using a two-tailed paired Student’s *t*-test, whereas comparisons between two independent groups were performed using an unpaired two-tailed Student’s t-test, as appropriate. Associations between categorical variables were assessed using Fisher’s exact test for 2 × 2 contingency tables. For R × C contingency tables with small or sparse cell frequencies, the Fisher–Freeman–Halton exact test was applied. All statistical tests were two-sided, and a *P-value* < 0.05 was considered statistically significant. Data are presented as mean ± SD or mean ± SEM, where applicable, in the corresponding figure legends.

## Results

### Clinical characteristics of the included patients

This study included 28 female patients with TNBC. The mean age of the patients was 48.7 ± 11.2 years. Twelve patients (42.9%) were premenopausal (aged >50 years), while 16 (57.1%) were postmenopausal (aged ≤50 years). Patients aged ≤50 years were considered younger women for the purpose of this study. Most patients (22/28, 78.6%) presented with advanced clinical stage (stage III–IV), whereas 6 (21.4%) had stage I–II disease. The clinicopathological characteristics of the study population are summarized in **Table 1**.

### Upregulated*TRIM33* expression in TNBC patients

Quantitative real-time PCR analysis showed increased *TRIM33* mRNA expression in TNBC tissues compared that to in paired adjacent non-tumor breast tissues. Increased *TRIM33* mRNA expression was observed in 19 of 28 cases (67.9%), whereas three cases (10.7%) showed decreased expression and six cases (21.4%) showed no appreciable change in expression. The mean relative expression of *TRIM33* was higher in tumor tissues than in the corresponding adjacent nontumor tissues. The association between *TRIM33* mRNA expression and the clinicopathological characteristics of the patients is summarized in **Figure 1** and **Table 2**.

**Figure 1:**
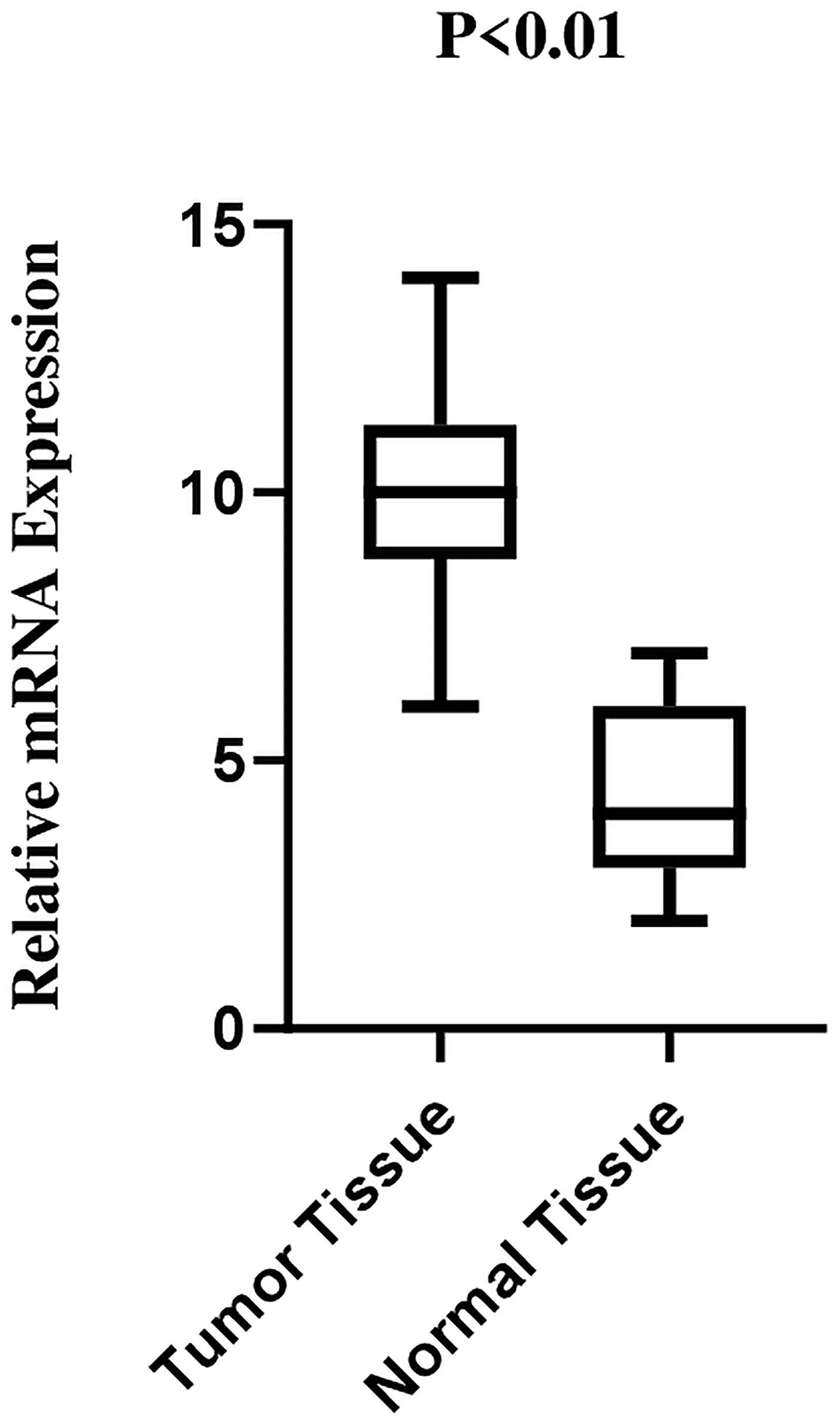
*TRIM33* mRNA expression in paired TNBC tumor and adjacent non-tumor breast tissues. Relative *TRIM33* mRNA expression was determined by quantitative reverse-transcription PCR (qRT-PCR) using *GAPDH* as the endogenous control and calculated using the 2^−ΔΔCt^ method, with adjacent non-tumor tissue used as the calibrator. Each pair represents tumor and corresponding adjacent non-tumor tissue from the same patient (n = 28 pairs). Statistical analysis was performed on ΔCt values using a two-tailed paired Student’s *t*-test. *P* = 0.0045.

**Table 2:** *TRIM33* mRNA expression according to clinicopathological characteristics of patients with triple-negative breast cancer.

Characteristics	n (%)	Relative *TRIM33* mRNA expression, mean ± SE	*P* value
Age (years)			
≤50	16 (57.10)	3.2 ± 0.27	0.032*
>50	12 (42.90)	2.3 ± 0.29	
Menopausal status			
Premenopausal	12 (42.90%)	3.50 ± 0.18	0.761
Postmenopausal	16 (57.10%)	3.60 ± 0.27	
Lymph-node status			
Negative	05 (17.86%)	2.10 ± 0.25	0.170
Positive	23(82.14%)	2.70 ± 0.34	
Tumor size (cm)			
≤5	05 (17.86%)	2.10 ± 0.02	0.004*
>5	23 (82.14%)	0.32 ± 0.55	
Clinical stage			
III–IV	22(78.57%)	2.5 ± 0.43	0.148
I–II	06(21.43%)	3.4 ± 0.41	

Clinicopathological data are presented as *n* (%), and relative *TRIM33* mRNA expression is presented as mean ± standard error (SE). Relative *TRIM33* mRNA expression was determined using the 2^−ΔΔCt^ method, with adjacent non-tumor tissue used as the calibrator and *GAPDH* as the endogenous control. Comparisons between two independent groups were performed using an unpaired two-tailed Student’s *t*-test, as appropriate. A two-sided *P* < 0.05 was considered statistically significant. An asterisk (*) indicates a statistically significant difference (P < 0.05).

### Expression of the TRIM33 protein and its correlation with clinicopathological characteristics in TNBC

Western blotting and immunohistochemistry were used to evaluate TRIM33 protein expression in TNBC tissues. Elevated TRIM33 protein expression was observed in 16 of 28 cases (57.1%). Overall, TRIM33 protein expression was higher in tumor tissues than in adjacent non-tumor breast tissues. The association between TRIM33 protein expression and clinicopathological characteristics is summarized in **Table 3**. Representative western blot and immunohistochemical findings are shown in **Figures 2**, **3**, respectively.

**Table 3:** Association of TRIM33 protein expression with clinicopathological characteristics of patients (n = 28).

Characteristics	n (%)	High *TRIM33*, n	Low *TRIM33*, n	P value
Age (years)				
≤50	16 (57.1)	6	10	0.023*
>50	12 (42.9)	10	2	
Menopausal status				
Premenopausal	12 (42.9)	10	2	0.023*
Postmenopausal	16 (57.1)	6	10	
Lymph node status				
Negative	05 (17.86)	4	1	0.355
Positive	23 (82.14)	12	11	
Tumor size				
≤5 cm	05 (17.86%)	2	3	0.354
>5 cm	23 (82.14%)	15	8	
Clinical stage				
III–IV	22 (78.6)	16	6	0.002*
I–II	6 (21.4)	0	6	

*TRIM33* protein expression was evaluated by immunohistochemistry and categorized as high or low according to the predefined scoring criteria. Data are presented as *n* (%). Associations between *TRIM33* protein expression categories and categorical clinicopathological variables were assessed using the two-sided Fisher–Freeman–Halton exact test, as appropriate. A two-sided *P* < 0.05 was considered statistically significant. *indicates *P* < 0.05. Bold values indicate statistically significant associations.

**Figure 2:**
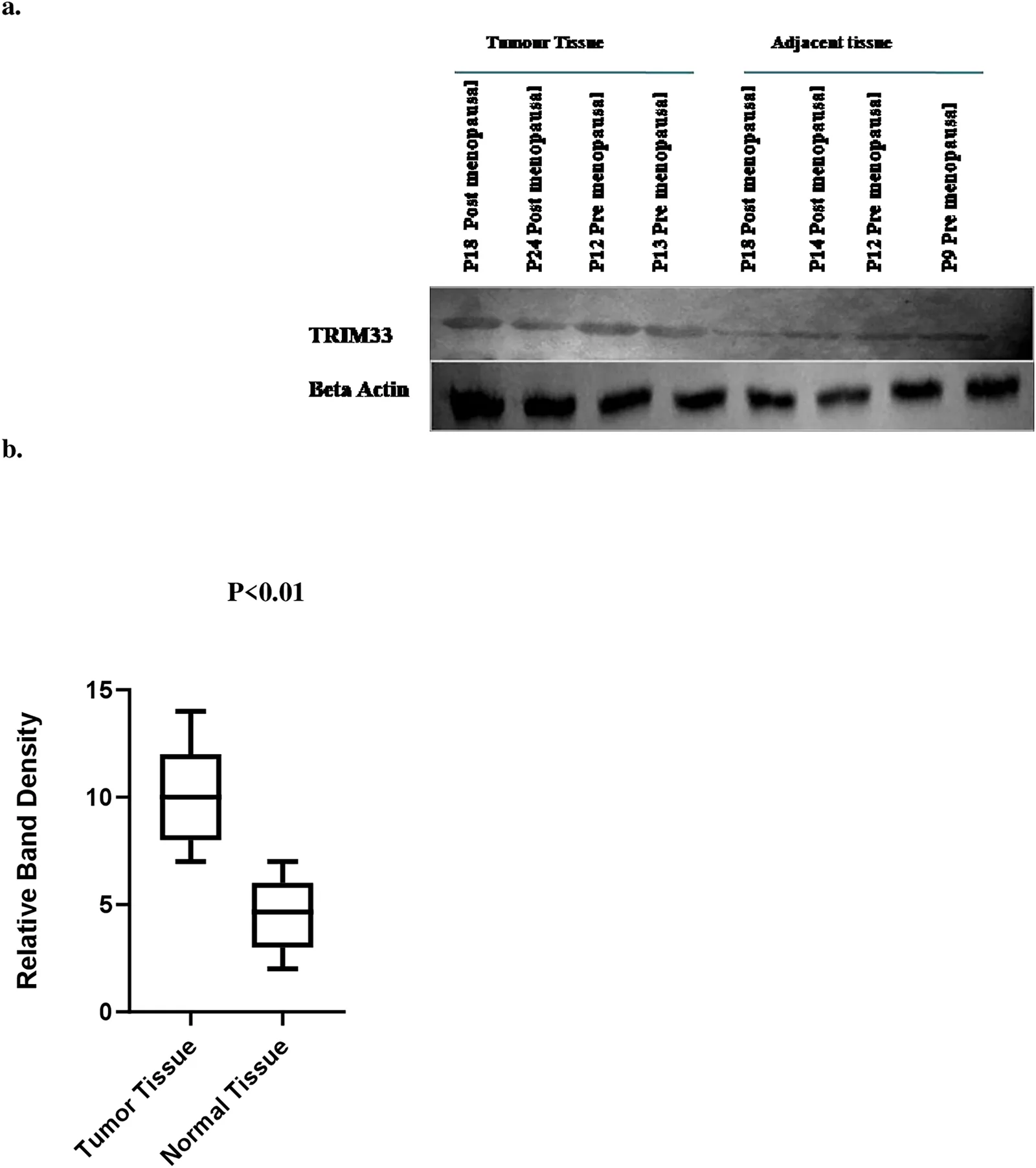
*TRIM33* protein expression in paired TNBC tumor and adjacent non-tumor breast tissues assessed by western blotting. **(a)** Representative Western blot showing *TRIM33* protein expression in tumor and corresponding adjacent non-tumor breast tissue. **(b)** Relative *TRIM33* protein expression was quantified by densitometric analysis and normalized to *GAPDH* in matched tumor and adjacent non-tumor tissues. Data from 28 matched biological samples are shown (n = 28 pairs). Statistical significance was assessed using a two-tailed paired Student’s *t*-test. A two-sided *P* < 0.05 was considered statistically significant.

**Figure 3:**
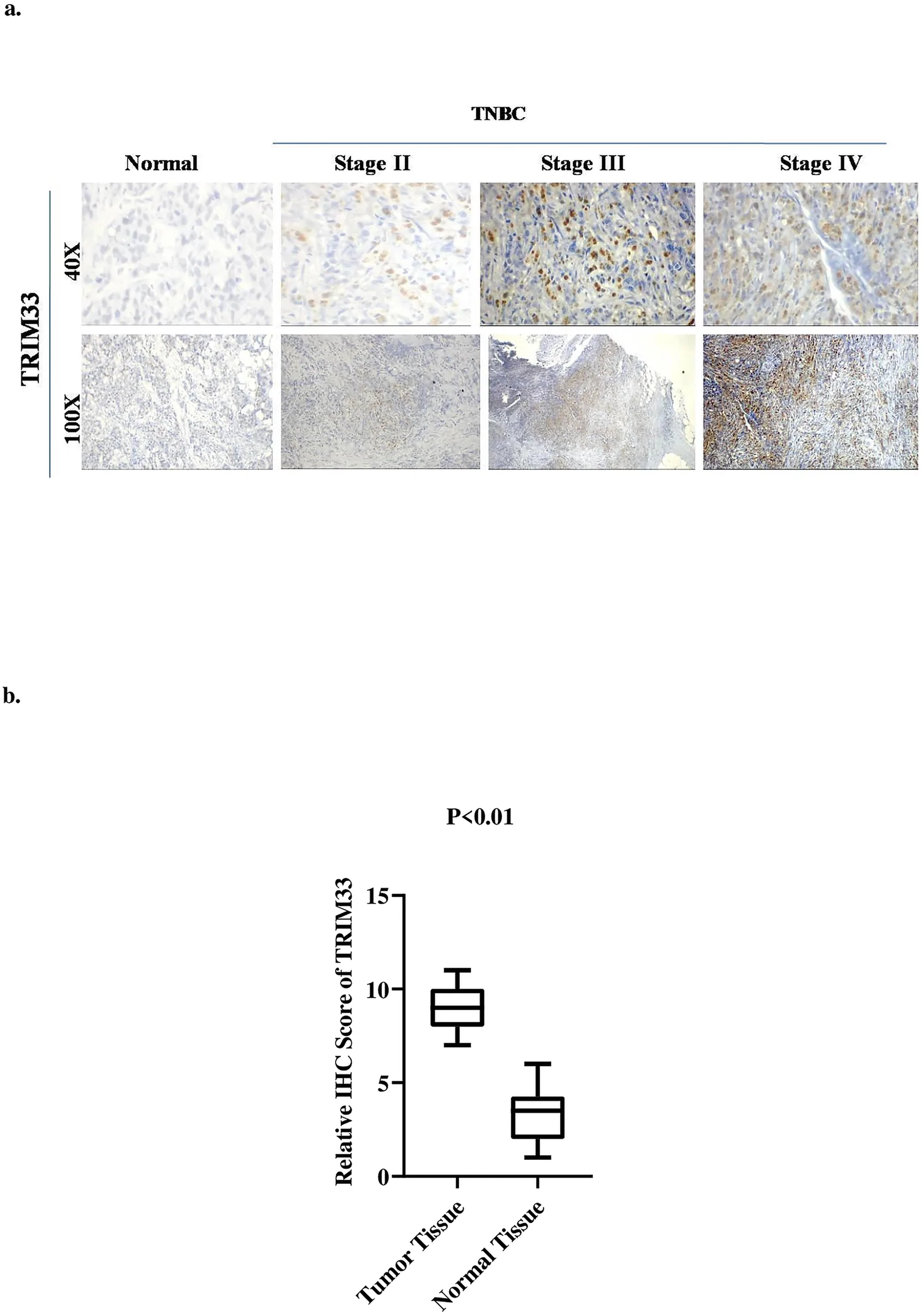
*TRIM33* protein expression in TNBC tumor and paired adjacent non-tumor breast tissues assessed by immunohistochemistry (IHC). **(a)** Representative IHC staining showing *TRIM33* protein expression in TNBC tumor tissues and their corresponding paired adjacent non-tumor tissues across the indicated clinical stages. **(b)** Quantitative comparison of *TRIM33* IHC scores between paired tumor and adjacent non-tumor tissues. Data are presented for 28 independent paired biological samples (n = 28 pairs). Statistical significance was assessed using a two-tailed paired Student’s *t*-test. A two-sided *P* < 0.05 was considered statistically significant.

### Promoter hypomethylation in cases with *TRIM33* overexpression

Methylation-specific PCR was used to evaluate the methylation status of the *TRIM33* promoter. An unmethylated/hypomethylated promoter pattern was observed in 22 of 28 cases (78.6%), whereas 3 cases (10.7%) showed a methylated pattern and 3 cases (10.7%) were classified as unaltered. The unmethylated/hypomethylated promoter pattern was frequently observed among cases showing increased *TRIM33* expression. Promoter methylation status was also associated with selected clinicopathological characteristics, including clinical stage, as summarized in **Table 4**. Representative MS-PCR findings are shown in **Figure 4**.

**Table 4:** Association of *TRIM33* promoter methylation status with clinicopathological characteristics of patients with triple-negative breast cancer (n = 28).

Characteristics	n (%)	*TRIM33* methylated, n	*TRIM33* unmethylated/hypomethylated, n	*TRIM33* unaltered, n	*P* value
Age (years)					
≤50	16 (57.14%)	01	13	02	0.809
>50	12 (42.86%)	02	09	01	
Menopausal status					
Premenopausal	12 (42.86%)	3	6	3	0.002*
Postmenopausal	16 (57.14%)	0	16	0	
Lymph-node status					
Negative	05 (17.86%)	01	02	02	0.050
Positive	23 (82.14%)	02	20	01	
Tumor size (cm)					
≤5	05 (17.86%)	01	02	02	0.066
>5	23 (82.14%)	01	20	02	
Clinical stage					
III–IV	22 (78.57%)	0	22	0	<0.001*
I–II	6 (21.43%)	3	0	3	

*TRIM33* promoter methylation status was determined by methylation-specific PCR (MS-PCR) and categorized according to the predefined methylation criteria. Data are presented as *n* (%). Associations between *TRIM33* promoter methylation status and categorical clinicopathological variables were assessed using the two-sided Fisher–Freeman–Halton exact test, as appropriate. A two-sided *P* < 0.05 was considered statistically significant. *indicates *P* < 0.05. An asterisk (*) indicates a statistically significant difference (P < 0.05).

**Figure 4:**
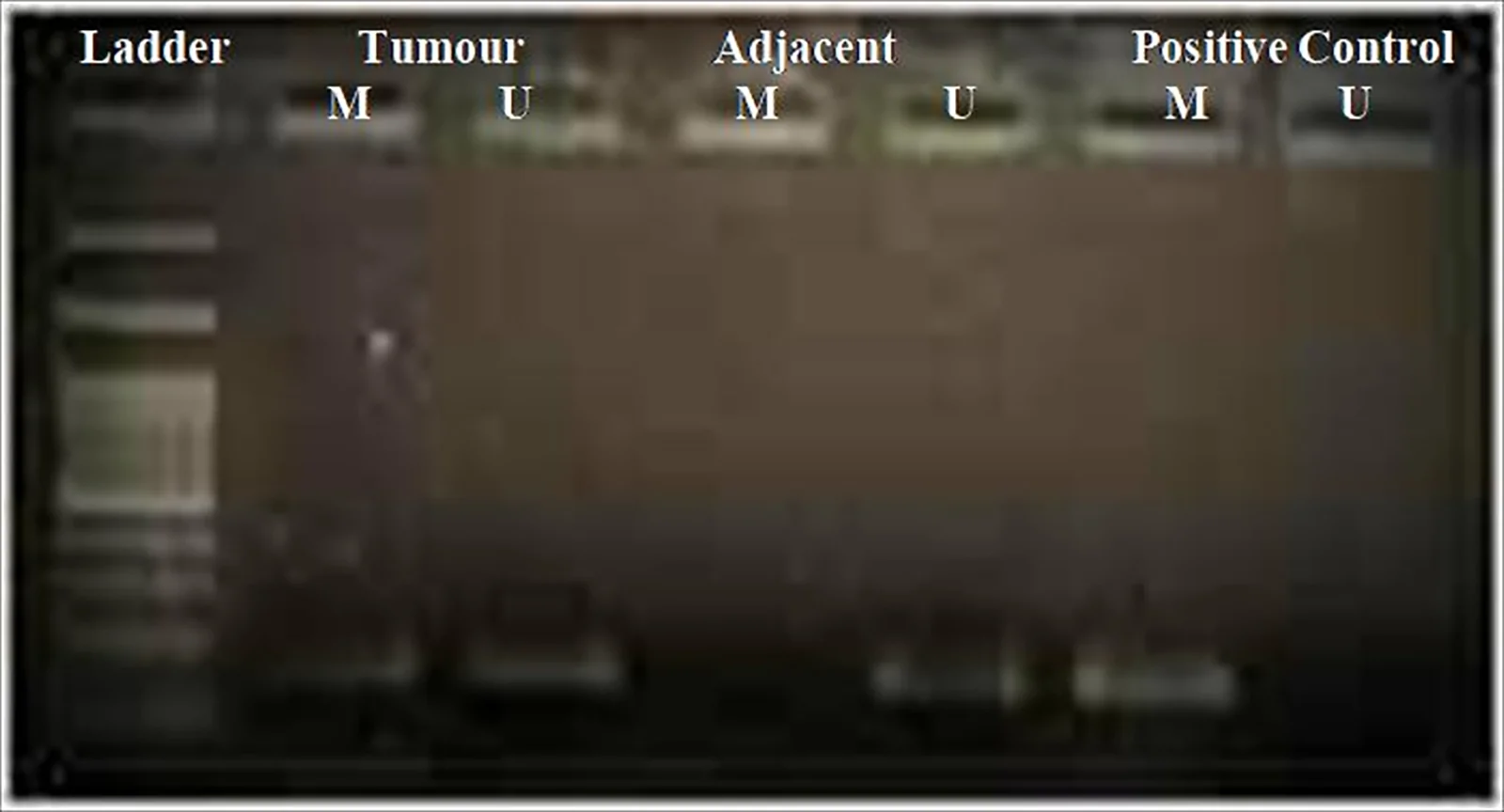
Representative agarose gel electrophoresis showing *TRIM33* promoter methylation status determined by methylation-specific PCR (MS-PCR). Representative MS-PCR results showing methylated (M; 170 bp) and unmethylated (UM; 171 bp) *TRIM33* promoter-specific PCR products in paired TNBC tumor and adjacent non-tumor breast tissues. The 100-bp DNA molecular-weight ladder is shown in lane 1. A total of 28 paired tumor and adjacent non-tumor tissue samples were analyzed (n = 28 pairs). The presence or absence of methylated- and unmethylated-specific PCR products was used for qualitative assessment of *TRIM33* promoter methylation status.

## Discussion

The tripartite motif (TRIM) family of proteins is involved in several cellular processes, including ubiquitination, transcriptional regulation, autophagy, innate immunity, apoptosis, and cell proliferation. The dysregulation of TRIM family proteins has been implicated in the development and progression of several malignancies (8, 15). Among these proteins, *TRIM33* has attracted particular interest because its role in cancer appears to be context-dependent, with both tumor-suppressive and tumor-promoting functions reported in different malignancies (11, 15). *TRIM33* modulates transcriptional activity and protein stability and interacts with signaling pathways relevant to tumor biology (15). Previous studies have reported the tumor-suppressive functions of *TRIM33* in several malignancies, whereas tumor-promoting effects have also been described in specific molecular contexts (16, 17). These contrasting observations suggest that the biological effects of *TRIM33* may depend on the tumor type and the molecular pathways operating within the tumor microenvironment.

In the present study, we evaluated *TRIM33* expression and promoter methylation status in 28 patients with TNBC. Increased *TRIM33* mRNA expression was observed in 19 of 28 cases (67.9%), while elevated protein expression was detected in 16 of 28 cases (57.1%). In addition, an unmethylated/hypomethylated *TRIM33* promoter pattern was identified in 22 of 28 cases (78.6%). These findings indicate that altered *TRIM33* expression and promoter methylation are frequent molecular events in TNBC samples examined in this study.

An important observation in the present study was the association between elevated *TRIM33* expression and advanced clinical stage. TRIM33 protein expression was significantly associated with clinical stage, with increased expression occurring more frequently in patients with advanced-stage disease. This finding suggests that *TRIM33* dysregulation may be related to disease progression in TNBC. However, because the present study was cross-sectional and did not include longitudinal survival analysis, this association should not be interpreted as direct evidence of a prognostic effect. Previous studies on other malignancies have also demonstrated associations between altered *TRIM33* expression and clinicopathological characteristics, although the direction of these associations varies according to tumor type and molecular context (11, 15, 18).

In the present study, elevated TRIM33 protein expression was observed in 16 of 28 patients (57.1%), whereas increased *TRIM33* mRNA expression was detected in 19 of 28 patients (67.9%). The difference between mRNA and protein expression may reflect post-transcriptional and post-translational regulatory mechanisms, including variations in mRNA stability, translational efficiency, protein stability, and protein degradation. Nevertheless, an overall pattern of increased *TRIM33* expression was observed at both the mRNA and protein levels. Previous studies have implicated *TRIM33* in the maintenance of genomic stability and cellular responses to DNA damage. In particular, *TRIM33* has been shown to participate in the PARP-dependent DNA-damage response through its interaction with *ALC1* (19, 20). However, the mechanisms linking *TRIM33* transcription, protein expression, and epigenetic regulation in TNBC remain to be fully elucidated.

Thus, any epigenetic alterations that might be linked to the aberrant expression of this gene in TNBC were investigated using methylation-specific PCR (MS-PCR).

Methylation-specific PCR revealed an unmethylated/hypomethylated *TRIM33* promoter pattern in 22 of 28 TNBC cases (78.6%). The association between *TRIM33* promoter methylation status and clinicopathological characteristics is summarized in **Table 4**. The frequent occurrence of unmethylated/hypomethylated promoter patterns alongside increased *TRIM33* expression suggests a possible relationship between promoter methylation status and *TRIM33* regulation in TNBC. Promoter hypomethylation may facilitate transcriptional activation; however, our findings demonstrate an association and do not establish a causal relationship between promoter methylation and increased *TRIM33* expression. Furthermore, MS-PCR provides a qualitative assessment of methylation status and does not quantify methylation at specific CpG sites. Therefore, future studies using quantitative approaches, such as pyrosequencing or targeted bisulphite sequencing, in larger patient cohorts would be valuable for confirming and characterizing CpG-specific methylation patterns within the *TRIM33* promoter.

The context-dependent role of *TRIM33* is further supported by findings from studies on other malignancies. Increased *TRIM33* expression has been reported in esophageal squamous cell carcinoma, and experimental studies have demonstrated that *TRIM33* promotes tumor growth and alters cellular metabolism (11). In contrast, reduced *TRIM33* expression is associated with tumor progression and adverse clinical outcomes in hepatocellular carcinoma, supporting a tumor-suppressive role for *TRIM33* in this setting (21). Taken together, these observations suggest that the biological consequences of *TRIM33* dysregulation may vary according to tumor type, molecular background, and signaling pathways involved. Therefore, the increased *TRIM33* expression observed in the present TNBC cohort should be interpreted within this context rather than as evidence of a universally oncogenic role for *TRIM33.*

Age and menopausal status may influence breast cancer biology through changes in inflammatory and immune signaling. Previous studies have reported associations between circulating inflammatory markers and postmenopausal breast cancer risk (22). In addition, preclinical studies on TNBC have demonstrated age-related differences in immune responses, with older mice showing a reduced response to immune checkpoint blockade compared to younger mice (23). Local inflammatory changes have also been associated with age-related lobular involution of breast tissue (24). Collectively, these findings suggest that age- and menopause-related alterations in inflammatory and immune signaling may influence the breast tumor microenvironment. However, the present study was not designed to investigate these mechanisms in relation to *TRIM33* expression. Therefore, further studies are required to determine how promoter methylation, genetic alterations, and other regulatory mechanisms contribute to altered *TRIM33* expression in TNBC and to clarify its potential clinical significance.

### Limitations of the study

The findings of the present study provide a basis for further investigation of *TRIM33* expression and promoter methylation in TNBC. The study was conducted in a relatively small cohort, and validation in larger and more diverse patient populations would help strengthen the generalizability of these findings. Future studies should investigate *TRIM33* methylation in peripheral blood or other body fluids in larger independent cohorts to determine whether it can serve as a minimally invasive diagnostic biomarker. As this study was cross-sectional, future prospective studies with clinical follow-up are needed to further clarify the relationship between *TRIM33* alterations and long-term clinical outcomes. In addition, MS-PCR provides useful information on promoter methylation status; however, quantitative approaches, such as pyrosequencing or targeted bisulfite sequencing, could provide more detailed information on methylation at individual CpG sites. Functional studies would be valuable for determining the biological relationship between promoter hypomethylation and increased *TRIM33* expression. Thus, our findings provide a foundation for larger prospective studies incorporating quantitative methylation analysis, functional validation, and long-term clinical follow-up.

## Conclusion

The present study demonstrated increased *TRIM33* expression at both the mRNA and protein levels in a substantial proportion of TNBC cases. An unmethylated/hypomethylated *TRIM33* promoter pattern was also frequently observed, suggesting a possible relationship between promoter methylation status and altered *TRIM33* expression as shown in graphical abstract **Figure 5**. In addition, *TRIM33* expression and promoter methylation status were associated with selected clinicopathological characteristics, particularly the clinical stage. These findings suggest that epigenetic regulation of *TRIM33* may play a role in the molecular biology of TNBC. However, considering the relatively small sample size and cross-sectional design of the study, these findings should be interpreted with caution. Further studies involving larger patient cohorts, quantitative methylation analysis, functional validation, and clinical follow-up are needed to clarify the biological and potential clinical significance of *TRIM33* dysregulation in TNBC.

**Figure 5:**
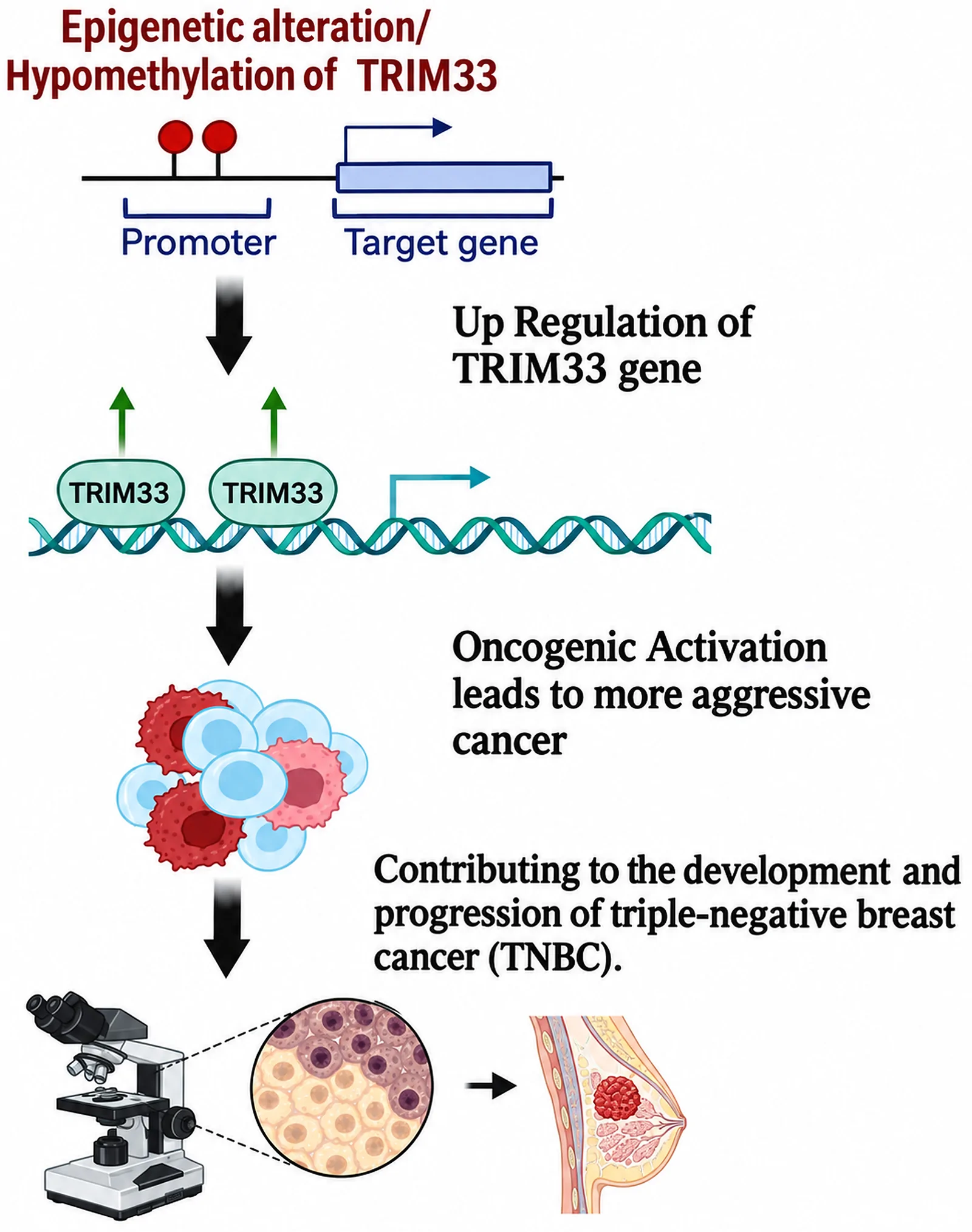
Schematic representation of the observed association between *TRIM33* promoter methylation and *TRIM33* expression in TNBC. The observed promoter hypomethylation was associated with increased *TRIM33* mRNA and protein expression in the analyzed TNBC tissue samples. This association supports a potential relationship between promoter methylation and *TRIM33* expression.

## Data Availability

The datasets presented in this study can be found in online repositories. The names of the repository/repositories and accession number(s) can be found in the article/**Supplementary Material**.

## References

[1] Łukasiewicz S Czeczelewski M Forma A Baj J Sitarz R Stanisławek A . Breast cancer-epidemiology, risk factors, classification, prognostic markers, and current treatment strategies-an updated review. Cancers. (2021) 13:4287. doi: 10.3390/cancers13174287 34503097 PMC8428369

[2] Xiong N Wu H Yu Z . Advancements and challenges in triple-negative breast cancer: a comprehensive review of therapeutic and diagnostic strategies. Front Oncol. (2024) 14:1405491. doi: 10.3389/fonc.2024.1405491 38863622 PMC11165151

[3] Chen Z Liu Y Lyu M Chan CH Sun M Yang X . Classifications of triple-negative breast cancer: insights and current therapeutic approaches. Cell Bioscience. (2025) 15:13. doi: 10.1186/s13578-025-01359-0 39893480 PMC11787746

[4] Dudley-Fraser J Rittinger K . It's a TRIM-endous view from the top: the varied roles of tripartite motif proteins in brain development and disease. Front Mol Neurosci. (2023) 16:1287257. doi: 10.3389/fnmol.2023.1287257 38115822 PMC10728303

[5] Hatakeyama S . TRIM family proteins: roles in autophagy, immunity, and carcinogenesis. Trends Biochem Sci. (2017) 42:297–311. doi: 10.1016/j.tibs.2017.01.002 28118948

[6] Romo BA Karakyriakou B Cressey L Brauer BL Yang H Warren A . TRIM33 is a co-regulator of estrogen receptor alpha. Cancers. (2024) 16:845. doi: 10.3390/cancers16050845 38473207 PMC10930732

[7] Gu M Ren B Fang Y Ren J Liu X Wang X . Epigenetic regulation in cancer. MedComm. (2024) 5:e495. doi: 10.1002/mco2.495 38374872 PMC10876210

[8] Huang N Sun X Li P Liu X Zhang X Chen Q . TRIM family contribute to tumorigenesis, cancer development, and drug resistance. Exp Hematol Oncol. (2022) 11:75. doi: 10.1186/s40164-022-00322-w 36261847 PMC9583506

[9] Wang F Wang W Wu X Tang C Du F Lu Z . Downregulation of TRIM33 promotes survival and epithelial-mesenchymal transition in gastric cancer. Technol Cancer Res Treat. (2022) 21:15330338221114505. doi: 10.1177/15330338221114505 35929141 PMC9358585

[10] Lee HJ . The role of tripartite motif family proteins in TGF-β signaling pathway and cancer. J Cancer Prev. (2018) 23:162–9. doi: 10.15430/JCP.2018.23.4.162 30671398 PMC6330992

[11] Xia T Meng L Xu G Sun H Chen H . TRIM33 promotes glycolysis through regulating P53 K48-linked ubiquitination to promote esophageal squamous cell carcinoma growth. Cell Death Dis. (2024) 15:740. doi: 10.1038/s41419-024-07137-z 39389957 PMC11467421

[12] Eberhardt W Haeussler K Nasrullah U Pfeilschifter J . Multifaceted roles of TRIM proteins in colorectal carcinoma. Int J Mol Sci. (2020) 21:7532. doi: 10.3390/ijms21207532 33066016 PMC7590211

[13] Jain S Singhal S Francis F Hajdu C Wang JH Suriawinata A . Association of overexpression of TIF1γ with colorectal carcinogenesis and advanced colorectal adenocarcinoma. World J Gastroenterol. (2011) 17:3994–4000. doi: 10.3748/wjg.v17.i35.3994 22046087 PMC3199557

[14] Zolota V Tzelepi V Piperigkou Z Kourea H Papakonstantinou E Argentou MI . Epigenetic alterations in triple-negative breast cancer-the critical role of extracellular matrix. Cancers. (2021) 13:713. doi: 10.3390/cancers13040713 33572395 PMC7916242

[15] Hatakeyama S . TRIM proteins and cancer. Nat Rev Cancer. (2011) 11:792–804. doi: 10.1038/nrc3139 21979307

[16] Ji W Zhang W Zhang X Ke Y . TRIM33 enhances the ubiquitination of TFRC to enhance the susceptibility of liver cancer cells to ferroptosis. Cell Signalling. (2024) 121:111268. doi: 10.1016/j.cellsig.2024.111268 38909931

[17] Eickhoff N Janetzko J Padrão N Gregoricchio S Siefert JC Hoekman L . TRIM33 loss reduces androgen receptor transcriptional output and H2BK120 ubiquitination. Commun Biol. (2025) 8:1043. doi: 10.1038/s42003-025-08449-2 40646276 PMC12254212

[18] Xu Y Wu G Zhang J Li J Ruan N Zhang J . TRIM33 overexpression inhibits the progression of clear cell renal cell carcinoma *in vivo* and *in vitro*. BioMed Res Int. (2020) 8:8409239. doi: 10.1155/2020/8409239 32908919 PMC7468622

[19] McAvera RM Morgan JJ Herrero AB Mills KI Crawford LJ . TRIM33 loss in multiple myeloma is associated with genomic instability and sensitivity to PARP inhibitors. Sci Rep. (2024) 14:8797. doi: 10.1038/s41598-024-58828-8 38627415 PMC11021562

[20] Kulkarni A Oza J Yao M Sohail H Ginjala V Tomas-Loba A . Tripartite motif-containing 33 (TRIM33) protein functions in the poly(ADP-ribose) polymerase (PARP)-dependent DNA damage response through interaction with amplified in liver cancer 1 (ALC1) protein. J Biol Chem. (2013) 288:32357–69. doi: 10.1074/jbc.M113.459164 23926104 PMC3820871

[21] Ding ZY Jin GN Wang W Chen WX Wu YH Ai X . Reduced expression of transcriptional intermediary factor 1 gamma promotes metastasis and indicates poor prognosis of hepatocellular carcinoma. Hepatol (Baltimore Md). (2014) 60:1620–36. doi: 10.1002/hep.27273 24954480

[22] Gunter MJ Wang T Cushman M Xue X Wassertheil-Smoller S Strickler HD . Circulating adipokines and inflammatory markers and postmenopausal breast cancer risk. J Natl Cancer Institute. (2015) 107:djv169. doi: 10.1093/jnci/djv169 26185195 PMC4651104

[23] Sceneay J Goreczny GJ Wilson K Morrow S DeCristo MJ Ubellacker JM . Interferon signaling is diminished with age and is associated with immune checkpoint blockade efficacy in triple-negative breast cancer. Cancer Discov. (2019) 9:1208–27. doi: 10.1158/2159-8290.CD-18-1454 31217296 PMC11167954

[24] Hanna M Dumas I Orain M Jacob S Têtu B Sanschagrin F . Association between local inflammation and breast tissue age-related lobular involution among premenopausal and postmenopausal breast cancer patients. PloS One. (2017) 12:e0183579. doi: 10.1371/journal.pone.0183579 28846716 PMC5573208

