# Supplementary material for: Promoter hypomethylation associated with elevated TRIM33 expression in triple-negative breast cancer patients
Source: Front Oncol. 2026 Oct 30;16:1973520. doi: 10.3389/fonc.2026.1973520 (PMC13759411; doi:10.3389/fonc.2026.1973520)
Supplement: Supplementary file 1 [file Supplementaryfile1.docx]

**Table 1 Details of *TRIM33* promoter coordinates, CpG sites, primer sequences used in the present study**

**From: Promoter Hypomethylation associated with elevated *TRIM33* expression in Triple-Negative Breast Cancer Patients**

**Fig-1**

**Fig. 1 Graphical representation of CpG islands in the *TRIM33* promoter region taken from Meth Primer.**

**Table 1. Primer setup**

| **Types** | **Gene** | **Primer sequence** | **Annealing temperature (°C)** | **Product size (bp)** |
| --- | --- | --- | --- | --- |
|  | ***TRIM33*** | **Methylation**  **F: 5’- TTTTACGGTTATTTAAAGGAACGC-3’**  **R: 5’- TAATTATAAACTCCGACACAACGAT -3’**  **Unmethylation**  **F:5’-TTTATGGTTATTTAAAGGAATGTGG -3’**  **R:5’-TAATTATAAACTCCAACACAACAAT -3’**  **RT-PCR**  ***TRIM33***  **F 5’** CCTCAGTTACCAATCCAGAAAACC**-3’**  **R 5’ -** AGCATCTTCCAACTGTATGGGT**-3’**  ***Beta Actin***  **F5’ -TCACCCACACTGTGCCCATCTACGA-3’**  **R 5’-CAGCGGAACCGCTCATTGCCAATGG-3’** | **49**  **56**  **54**  **58** | **170**  **171**  **248**  **452** |

**Table 1: Table showing primer sequences used for methylation analysis along with primer used in real-time PCR to quantify the expression of *TRIM33* gene.**
